# Poor-Law Medical Officers and *Post-Mortems*

**Published:** 1914-06-06

**Authors:** J. Corker

**Affiliations:** Union Infirmary, Rochdale


### Poor-Law Medical Officers and Post-Mortems.

*To the Editor of* The Hospital.

Sir,—An experience I had a few days ago is, I think, rather a good instance of how one branch of the medical profession is exploited at the present time.

As resident medical officer of this institution I admitted a few days ago a patient who had a scalp wound and was suffering from erysipelas of head, neck, and face and bronchitis. About two days after admission the patient died and I notified his death to the coroner, who, after making inquiries as to the cause of the scalp wound, decided to hold an inquest and instructed me to make a *post-mortem* examination. On my stating that I was quite satisfied as to the cause of death without making a *post-mortem*, he said one must be made as the relatives of deceased were going in for compensation. I, of course, made the *post-mortem*, but felt that I was being most unconscionably put on, not by the coroner, who has shown himself at all times most considerate, but by the law as it now stands, which compels me to give my time, my services, and my knowledge free of charge for the benefit of the parties concerned in a compensation claim.

It is high time, I think, that some steps were taken to get this most unjust law altered.—Yours truly,

J. Corker.

Union Infirmary, Rochdale, May 28, 1914.

